# Surgical outcome of unilateral recess-resect in convergence insufficiency-type intermittent exotropia: Comparison of two surgical formulae

**DOI:** 10.1371/journal.pone.0343366

**Published:** 2026-03-20

**Authors:** Hye Jun Joo, Hyeon Jung Kim, Dong Gyu Choi

**Affiliations:** 1 Department of Ophthalmology, Seoul National University Hospital, Seoul, South Korea; 2 Department of Ophthalmology, Kangnam Sacred Heart Hospital, Hallym University College of Medicine, Seoul, South Korea; Hangil Eye Hospital / Catholic Kwandong University College of Medicine, KOREA, REPUBLIC OF

## Abstract

**Aim:**

We compared the surgical outcomes of monocular lateral rectus recession–medial rectus resection (RR) for convergence insufficiency-type intermittent exotropia (CI-type IXT) using two different formulas.

**Methods:**

A retrospective review of patients who underwent unilateral RR for CI-type IXT with at least 6 months follow-up was done. In group 1 (58 patients), RR was determined based on the average of distance and near exodeviation. In group 2 (80 patients), lateral rectus (LR) recession amount was based on the exodeviation at distance and medial rectus (MR) resection was done as the same amount of LR recession (symmetric RR).

**Results:**

Group 2 showed greater esodeviation at distance and near until postoperative 1 month, however, there was no significant difference in deviation angles between the two groups from 3 to 24 months. Both groups showed significant reduction in near-distance difference up to postoperative 24 months (p < 0.05): from 8.3 PD preoperatively to 1.0 at 24 months in group 1 and from 9.3 to 3.8 in group 2. The success rate at 24 months was 72.7% and 67.6% in group 1 and 2, respectively (p = 0.795).

**Conclusion:**

RR based on the average of distance and near exodeviation and the symmetric RR can successfully collapse the near–distance difference while achieving favorable surgical outcomes.

## Introduction

Conventionally, intermittent exotropia (IXT) has been categorized into four groups based on the distance and near deviation as proposed by Burian [1]. Of them, convergence insufficiency-type (CI-type) IXT was defined as when the near deviation exceeded 5–15 prism diopters (PD) or more than the distance deviation [2–4].

Unilateral lateral rectus recession and medial rectus resection (RR) is a common surgical technique for treating CI-type IXT, with several formulae proposed to determine the appropriate amount of resection and/or recession. However, each method has its own set of advantages and disadvantages. Many surgeons have aimed to correct exodeviation at near fixation as the desired surgical goal for CI-type IXT, but carries the risk of distant diplopia due to overcorrection at distance for a significant period after surgery. On the other hand, Kushner suggested to correct the distance deviation, which would leave the near improved, but undercorrected. He managed the improved exodeviation at near with prisms if it was symptomatic [5]. To address this near-distance disparity, Kraft et al. [6] proposed a technique in which the strengthening of medial rectus (MR) exceeds the weakening of lateral rectus (LR), with MR resection based on near deviation and LR recession based on distance. Choi et al. [7] also reported that MR strengthening more than LR weakening in children with CI-type IXT successfully reduced both distance and near deviation and collapsed near-distance differences. This approach has since become one of the most widely adopted surgical strategies for CI-type IXT. However, according to this formula, for example, a patient with 20 PD at distance and 40 PD at near would undergo a 5.0 mm LR recession and a 7.0 mm MR resection. Consequently, the amount of MR resection is considerably greater than the amount of LR recession, which may in turn increase the potential risk of complications related to over-resection as well as overcorrection, especially at distance.

From an anatomical perspective, the MR has the shortest arc of contact to the sclera (6 mm) and the LR the longest (10 mm) among four rectus muscles because of the divergent angle of the orbit versus the visual axis. Moreover, the rectus muscle insertions form a progressive spiral (spiral of Tillaux) around the limbus with the MR being closer to the limbus (5–5.5 mm) than the LR (7 mm). Therefore, performing a greater amount of MR strengthening compared to LR weakening might cause imbalance between MR and LR.

To address this concern and achieve a balance in correcting both distance and near deviations without excessively increasing MR resection relative to LR recession, two modified surgical approaches were implemented. The purpose of this study was to compare the surgical outcomes of two surgical formulae in unilateral RR for CI-type IXT; based on the average of the distance and near angle, and the amount of LR recession based on distance deviation and MR resection on the same amount as LR recession (symmetric RR).

## Materials and methods

This study was conducted in accordance with the Declaration of Helsinki, and the requirement for informed consent was waived owing to the study’s retrospective nature, and ethical approval was granted by the Institutional Review Board of Hallym University Medical Center (IRB No. 2023-05-033). All data were accessed for research purposes between 01/11/2023 and 31/05/2024. The dataset we used did not contain any personally identifiable information.

### Subject recruitment

In this retrospective study, medical records of 138 patients who had undergone RR procedure for CI-type IXT with minimum 6-month postoperative follow-up periods were reviewed. The CI-type IXT was classified if exodeviation at near fixation was greater by 10 PD (prism diopters) or more compared to that at distant fixation in patients with near deviations ≥ 30 PD, however, by one-third of the near deviation in patients with near deviations < 30 PD [2,8] because 10 PD would be a relatively significant difference to analyze the surgical outcomes and the postoperative reductions of near-distance (N-D) difference in the small angle exodeviation. Patients with ophthalmic diseases other than strabismus, a history of previous strabismus surgery, paralytic or restrictive strabismus, or systemic diseases such as Down syndrome or cerebral palsy were excluded from this study.

### Preoperative evaluation

We noted the preoperative characteristics of subjects including age at onset and surgery, gender, exodeviation at distance and near, presence of lateral incomitance associated strabismus such as dissociated vertical deviation (DVD) vertical deviation, or oblique muscle dysfunction, and sensory status. All patients underwent full ophthalmological evaluations before surgery with their best optical correction based on cycloplegic refraction. The angle of deviation was measured with the alternate prism cover test at distance (6 m) and near (33 cm). We defined lateral incomitance as exodeviation decrease of 20% or 5 PD or more at lateral gaze compared to the primary position, and vertical deviation as hyper- or hypotropia more than 5 PD in the primary position. Sensory status evaluation was done using Titmus Stereotest (Stereo Optical Co., Inc., Chicago, IL, USA) at 40 cm distance and Worth-4-Dot test (Richmond Products, Albuquerque, NM, USA) at 6 meters. The stereoacuity of 70 seconds of arc or better using Titmus Stereotest was defined as “good stereopsis,” and the result of Worth 4-dot test as “fusion” if patients saw 4 dots.

### Surgery & grouping

All surgeries were performed under general anesthesia by a single surgeon (DGC), who had previously determined the amount of RR for CI-type IXT based on the average of the distance and near angles of exodeviation (group 1). However, recently, the amount of LR recession has been determined based on the distance deviation, with the MR resection augmented by an amount equal to the LR recession. This method is referred to as “symmetric RR” in this study (group 2). The surgical dosages are presented in Table 1.

**Table 1:** Surgical dosages of RR in group 1 and group 2.

Target angles (PD)^a^	Group 1^b^ LR recess (mm) / MR resect (mm)	Group 2^c^ LR recess (mm) / MR resect (mm)
**20**	5.0 / 4.0	5.0 / 5.0
**25**	6.0 / 5.0	6.0 / 6.0
**30**	7.0 / 5.5	7.0 / 7.0
**35**	7.5/ 6.5	7.5 / 7.5
**40**	8.0 / 7.0	8.0 / 8.0
**45**	8.5 / 7.0	
**50**	9.0 / 8.0	

RR = monocular lateral rectus recession–medial rectus resection; PD = prism diopters: LR = lateral rectus; MR = medial rectus.

^a^The average of the distance and near angles of exodeviation in group 1 and distance angles of exodeviation in group 2.

^b^Group 1: The amount of RR was based on the average of the distance and near angles of exodeviation.

^c^Group 2: The LR recession amount was based on the exodeviation at distance and MR resection was done on the same amount as LR recession (symmetric RR).

### Postoperative management

Alignment at distance and near fixation in the primary position was measured in all patients at 1week and 1, 3, 6, 12, and 24 months postoperatively and at their last follow-up visit. If the patient complained of diplopia or had esodeviation postoperatively, alternate full-time patching was prescribed until diplopia or esodeviation resolved. If the esodeviation was still unresolved at 2 months, refractive errors were re-corrected after cycloplegic refraction and /or base-out Fresnel press-on prisms (3 M Press-On Optics; 3 M Health Care, St Paul, MN, USA) were prescribed.

### Outcome measures

We compared the mean angle of deviation at distance and near, postoperative reduction in the N-D difference compared to the preoperative status, and the surgical success rates between group 1 and 2 at each follow-up visit. Surgical success was defined as an alignment between exodeviation of <10PD and esodeviation of <5PD at distance and near fixation. Exodeviation is represented as positive value, and esodeviation as negative.

### Statistical analysis

The Student t-test and Pearson chi-square test were used for the analysis. All statistical analyses were performed using SPSS software, version 26 (SPSS Inc, Chicago, IL). A statistically significant difference was considered if the P value was less than 0.05.

## Results

Among the 138 patients, 58 patients were in group 1 and 80 in group 2. The demographic characteristics of subjects in this study are shown in Table 2. The preoperative mean angle of deviation was not significantly different between group 1 and group 2 at both distance (p = 0.061) and near (*p = 0.231*) and the N-D difference between group 1(8.3 ± 3.6 PD) and 2 (9.3 ± 3.2) did not show statistical difference (*p = 0.091*). The mean follow-up period after the surgery was 71.0 ± 55.8months (range, 7–235 months) in group 1, and 25.5 ± 18.4 (range, 6–94) in group 2 (*p < 0.001*), which can be explained by the fact that the follow-up period of group 2 (symmetric RR) was shorter than that of group 1 because symmetric RR was recently conducted by the surgeon (DGC).

**Table 2:** Demographic data of subjects.

	Group 1 (n = 58)	Group 2 (n = 80)	*p-value*
**Age at surgery (year)**	9.8 ± 11.9	12.4 ± 11.6	0.194^a^
**Sex (male:female)**	23:35	37:43	0.440^b^
**Exodeviation (PD)**			
**at distance**	29.1 ± 9.7	26.2 ± 8.8	0.061 ^a^
**at near**	37.5 ± 10.9	35.4 ± 8.8	0.231^a^
**Preoperative near-distance difference (PD)**	8.3 ± 3.6	9.3 ± 3.2	0.091^a^
**Lateral incomitance [n (%)]**	6/58 (10.3%)	3/80 (3.8%)	0.121^b^
**Associated strabismus**			
**DVD [n (%)]**	2/58 (3.4%)	3/80 (3.8%)	0.941^b^
**Vertical deviation [n (%)]**	11/58 (19.0%)	11/80 (13.8%)	0.383^b^
**Oblique muscle dysfunction [n (%)]**	24/58 (41.4%)	16/80 (20%)	0.006^b^
**Preoperative sensory status**			
**Good stereopsis [n (%)]** **(70 arcsec or better in Titmus test)**	20/45 (44.4%)	48/78 (61.5%)	0.062^b^
**Fusion on Worth-4-dot test [n (%)]**	26/47 (55.3%)	42/71 (59.2%)	0.958^b^
**Postoperative follow-up duration (months)**	71.0 ± 55.8	25.5 ± 18.4	<0.001^a^

PD = prism diopters; DVD = dissociated vertical deviation.

Group 1: The amount of RR was based on the average of the distance and near angles of exodeviation.

Group 2: The LR recession amount was based on the exodeviation at distance and MR resection was done on the same amount as LR recession (symmetric RR).

^a^Student t-test; bPearson chi-square test.

At postoperative 1 week, the esodeviation was larger in group 2 (−3.7 ± 5.0) than group 1 (−0.7 ± 5.5) at distance (*p < 0.001*), however, not significantly different at near (−0.5 ± 5.5, −1.1 ± 4.4 in group 1 & 2, respectively, p = 0.495) (Table 3). There was no statistical difference in angles of deviation at distance and at near between the two groups from 3 months to 24 months. No case of overcorrection was observed in Group 1, whereas one case occurred in Group 2. The patient underwent an initial symmetric RR procedure of 8.0/8.0 mm for a preoperative deviation of 40 PD at distance and 50 PD at near. Secondary surgery was required at 1 year postoperatively to correct consecutive esotropia of 20 PD.

**Table 3:** Angles of exodeviation (PD) at distance and near in Group 1 and Group 2.

	Distance			Near		
	Group 1^a^ (n = 58)	Group 2^b^ (n = 80)	*p-value* ^c^	Group 1^a^ (n = 58)	Group 2^b^ (n = 80)	*p-value* ^c^
**Preoperative**	29.1 ± 9.7	26.2 ± 8.8	0.061	37.5 ± 10.9	35.4 ± 8.8	0.231
**Postoperative**						
**1 week**	−0.7 ± 5.5	−3.7 ± 5.0	<0.001	−0.5 ± 5.5	−1.1 ± 4.4	0.495
**1 month**	0.8 ± 3.8	−1.3 ± 3.4	<0.001	1.1 ± 3.4	0.5 ± 3.1	0.374
**3 months**	2.9 ± 6.1	0.6 ± 3.7	0.018	3.6 ± 7.4	1.8 ± 4.8	0.135
**6 months**	3.5 ± 6.2	1.7 ± 4.5	0.082	3.7 ± 7.7	4.5 ± 6.7	0.534
**12 months**	5.0 ± 7.3	3.1 ± 6.6	0.161	5.5 ± 9.1	6.3 ± 9.5	0.703
**24 months**	6.1 ± 7.0	3.7 ± 4.9	0.110	6.3 ± 7.6	7.5 ± 9.1	0.567
**Last follow-up**	9.0 ± 10.1	4.5 ± 7.5	<0.001	11.9 ± 11.6	8.2 ± 11.3	0.069

Mean ± SD.

PD = prism diopters.

^a^Group 1: The amount of RR was based on the average of the distance and near angles of exodeviation.

^b^Group 2: The LR recession amount was based on the exodeviation at distance and MR resection was done on the same amount as LR recession (symmetric RR).The positive value of deviation angles represented as exodeviation, and the negative value as esodeviation.

^c^Student t-test.

The N-D difference was notably reduced from preoperative values in both groups: from 8.3 PD preoperatively to 1.0 in group 1 and from 9.3 to 3.8 in group 2 at postoperative 24 months. However, this reduction in N-D difference showed no substantial difference between the two groups during follow-up (*p > 0.05*, Table 4). Additionally, the surgical success rate was not significantly different between the two groups (*p > 0.05*): 72.7% and 67.6% at postoperative 24 months in group 1 and 2, respectively (*p = 0.795*, Table 5).

**Table 4:** Postoperative reduction in Near-Far difference compared to preoperative values (PD).

	Group 1^a^ (n = 58)	Group 2^b^ (n = 80)	p-value^c^
**1 week**	7.9 ± 4.8	6.7 ± 4.2	0.210
**1 month**	7.8 ± 3.9	7.5 ± 4.1	0.770
**3 months**	7.5 ± 3.9	7.9 ± 3.6	0.531
**6 months**	7.7 ± 4.2	6.6 ± 4.8	0.258
**12 months**	6.8 ± 4.5	6.4 ± 4.8	0.867
**24 months**	7.1 ± 3.7	5.9 ± 6.3	0.241
**Last follow-up**	5.9 ± 4.9	5.6 ± 6.1	0.784

Mean ± SD.

PD = prism diopters.

^a^Group 1: The amount of RR was based on the average of the distance and near angles of exodeviation.

^b^Group 2: The LR recession amount was based on the exodeviation at distance and MR resection was done on the same amount as LR recession (symmetric RR).

^c^Student t-test.

**Table 5:** Surgical success rates (%).

	Group 1^a^ (n = 58)	Group 2^b^ (n = 80)	p-value^c^
**Postoperative**			
**1 month**	94.7	90.0	0.361
**3 months**	87.0	96.1	0.091
**6 months**	85.7	82.3	0.807
**12 months**	80.0	71.4	0.371
**24 months**	72.7	67.6	0.795
**Last follow-up**	50.0	62.5	0.165

Surgical success = the angle of deviation a distance and near between 10 PD exodeviation and 5 PD esodeviation.

^a^Group 1: based on the average of the distance and near angles of exodeviation.

^b^Group 2: based on distance deviation and MR resection on the same amount as LR recession (symmetric RR).

^c^Pearson chi-square test.

## Discussion

Our study evaluated the surgical outcome of a newly proposed surgical formula, the symmetric RR, in CI-type IXT. This method aimed to achieve a balance in correcting the near-distance disparity, while avoiding an excessive increase in MR resection. We found that symmetric RR was as effective as the RR formula based on the average of the distance and near angle in treating CI-type IXT in terms of collapse of the N-D difference and the surgical success rate.

The optimal reference value of N-D differences used in CI-type IXT classification remains debated, with Burian and Spivey [1,9] suggesting a 10PD difference, while Suh et al. [8] proposing a 10PD as the reference value in distance exodeviations exceeding 30 PD, but one-third of the distance deviation for exodeviations less than 30 PD, as a 10 PD difference would be relatively significant in smaller angles [2,8]. Our study used the classification proposed by Suh et al. [8] to accommodate patients with different degrees of exodeviation and avoid underestimating the impact of surgical treatments in small angles.

Previously, several surgical techniques have been proposed to reduce the near-distance disparity in CI-type IXT. Kraft et al. [6] first described in 1995 a technique of RR wherein the monocular strengthening of MR resection exceeds LR weakening; the amount of MR resection was based on near deviation, and the amount of LR recession on distance deviation. However, Farid reported high rates of postoperative undercorrection compared with other surgical techniques [10]. Wang et al. demonstrated that this RR technique achieved a better result than the unilateral (UMR) or bilateral MR (BMR) surgeries for patients with CI-type IXT [11]. However, since the UMR and BMR surgical formulae were based on the distance exodeviation, interpreting the results seems to require careful consideration. Archer [5] demonstrated that in cases of strabismus with unequal distance and near deviations, the perceived greater effect of MR surgery on near deviation and LR surgery on distance deviation was likely an artifact stemming from differences in preoperative characteristics. Therefore, whether a deviation is larger at distance or near fixation may not be a critical factor when deciding between MR or LR surgery [12]. Considering this aspect, the commonly used RR formula for CI-type IXT, which involves LR recession based on distance deviation and MR resection based on near deviation may lack sufficient evidence to support the validity of this formula.

The slanting procedures, in which the upper edge of the MR or LR was resected or recessed according to the distance exodeviation and the lower edge according to near exodeviation have been also tried [13,14]. However, this technique has been associated with vertical pattern strabismus, such as V and A patterns [10]. Despite the variety of surgical options and formulae for CI-type IXT, the optimal approach remains unclear, and reported outcomes are highly variable, with reported success rates varying widely from 18% to 92% [1,7,11–13,15,16].

In our study, the postoperative angle of deviation at distance was significantly smaller in the symmetric RR group until postoperative 1 month, but not significant different from postoperative 3 months to 24 months. Additionally, the N-D difference, which is crucial in CI-type IXT, showed a significant reduction compared to the preoperative status in both group 1 and group 2. The reduction in N-D difference did not show statistically significant difference between the two groups. The surgical success rate showed no significant differences between the two groups at any examined time point up to 24 months. Moreover, the surgical success rate at last follow-up was 50% in group 1 and 62.5% in group 2, which showed non-inferior results compared with previous different R&R formulae [7,11,12]. This suggests that the two surgical formulae used in this study are equally effective in helping the alignment of the eyes and collapsing the N-D difference in CI-type IXT.

According to the Parks table [17], the amount of MR resection performed during IXT surgery is generally smaller than the amount of LR recession. We presumed that it may possibly be due to orbital anatomical factors. The MR has the shortest arc of contact to the sclera (6 mm) and the LR the longest (10 mm) among four rectus muscles. The mechanical strength of an extraocular muscle is related to its length, arc of contact, and pulling direction [18]. Clark and Demer revealed through magnetic resonance imaging that MR after large resection for exotropia had a significantly larger angle at insertion than predicted from the “arc of contact” model. This finding suggests that increased extraocular muscle (EOM) bulk at the insertion from anterior shift of the EOM belly after surgical resection, substantially increases the angle at tendon insertion with subsequent loss of MR tangency to the globe [19]. Therefore, in cases with near-distance disparity, excessive MR resection targeted at near exodeviation angle may result in outcomes that differ from expectations. Moreover, excessive resection may mechanically restrict the movement of the globe in the opposite direction, and large resections may lead to globe retraction, resulting in narrowing of the palpebral fissure.

There are some limitations to our study. There were differences in the follow-up period between the two groups. The differing follow-up periods between the groups made direct comparisons of long-term outcomes challenging. While the shorter follow-up in symmetric RR group did not significantly skew the overall positive outcomes observed, longer-term studies are necessary to gain a more comprehensive understanding of the efficacy of these surgical approaches over time. Second, given the retrospective nature of the study, there might have been selection bias and lack of standardization. In addition, symmetric RR without surgical augmentation may be insufficient in cases with a pronounced distance–near disparity. Future studies incorporating longer, standardized follow-up periods and augmented surgical tables in selected cases with pronounced distance–near disparity are warranted to validate these findings.

Despite these limitations, our study is significant as it raised concerns that excessive strengthening of the MR compared to the weakening of the LR may be unphysiologic and lead to an imbalance between the two muscles. We introduced a new RR formula designed to prevent the amount of MR resection from exceeding that of LR recession by restricting the MR resection augmentation to match the amount of LR recession.

In conclusion, in CI-type IXT, the symmetric RR, which determines the amount of LR recession based on the distance angle and sets the MR resection amount symmetrically to the LR recession can successfully collapse the near–distance difference while achieving favorable surgical outcomes.
